# Thermal Exposure and Heat Illness Symptoms among Workers in Mara Gold Mine, Tanzania

**DOI:** 10.29024/aogh.2318

**Published:** 2018-08-31

**Authors:** E.B. Meshi, S.S. Kishinhi, S.H. Mamuya, M.G. Rusibamayila

**Affiliations:** 1 Department of Public Health, College of Health and Allied Sciences, The University of Dodoma, Dodoma, TZ; 2 Department of Environmental and Occupational Health, Muhimbili University of Health and Allied Sciences, Dar es Salaam, TZ; 3 Department of Epidemiology, Dodoma Regional Referral Hospital, Dodoma, TZ

## Abstract

**Background:**

Working in a humid and hot environment creates challenges to occupational health and safety in tropical countries. Being in the region, Tanzania can experiences more than 30°C ambient temperature, which exposes miners to heat-related injury and illness.

**Objectives:**

This study was conducted to assess heat stress exposure and associated heat illness symptoms among gold mine workers in the Mara region.

**Methods:**

A cross-sectional study was conducted among 60 miners from four Similar Exposure Groups based on risk to extreme heat environment. The WBGT index was used to assess the heat load while the miners’ physiological condition explained the heat strain indicator. Data was analyzed using SPSS 20. Chi-square was used to differentiate proportion of miners with heat illness symptoms in different categories. Pearson correlation was used to determine association between environmental measures and change in physiological conditions of the miners. Independent *t*-test and ANOVA were used to assess differences between numerical data among groups. A *p* ≤ 0.05 at 95% confidence was considered to be statistically significant.

**Findings:**

The recorded average WBGT at the mining site was within the ACGIH TLV of 28.5°C, however, 78.4% of underground miners and 69.6% of open cut miners reported to have moderate heat illness. High body temperature and hot and dry skin were the most frequently reported heat illness symptoms. The mean core body temperatures of miners in open cut and underground were 38.4 ± 0.5°C and 37.3 ± 0.5°C respectively. Approximately 80% of miners in open cut indicated higher core body temperature above ISO 7933 threshold of 38.0°C for safety. The majority of workers under contract didn’t drink water prior to work shift commencement.

**Conclusions:**

The occupational setting at the mining area presents the potential exposure to a thermal condition that can contribute to heat illness symptoms. Effective strategies must be implemented to enhance workers’ safety.

## Introduction

Tanzania is the fourth-largest gold miner in Africa behind South Africa, Mali and Ghana. The mining industry makes a significant contribution to the country economy [1]. It contributes to 0.5% of employment opportunities and is responsible for 3.3% of the Gross Domestic Product [2].Workers in the mining industry encounter are exposed to several hazards, heat stress being among them [3]. Heat stress as an aspect of the physical work environment directly affects human health, safety, and productivity and creates a significant economic burden in compensation claims [4].

The magnitude of heat stress and other heat-related illness has been experienced in different parts of the world. A study on heat strains, hydration status and symptoms of heat illness in 91 surface mine workers of Australia, found that 87% of workers reported at least one symptom over a 12-month period, with fatigue, headache and high body temperature being the most reported symptoms.

Because Tanzania is in a tropical region, with hot and humid temperatures, radiant heat energy from machines and equipment and workload severity increases the susceptibility of mine workers to heat stress injury and illness. Moreover, mine workers are equipped with PPE such as long-sleeved cotton jumpsuits, rubber or leather boots, hard hats with lamps, safety glasses, and self-contained-self-rescue apparatus that increase the insulation and prevent excessive heat loss [5]. To protect workers from thermal exposure, ILO, ISO 7933 and ACGIH have set measures, which include: limiting work permission when workers’ core body temperature exceeds 38°C, improving ventilation practices and promoting hydration practices [6,7,8].

Despite this, there are still reported cases of heat illness in the mining industry. The Mara region has an annual high ambient temperature of 28.4°C and relative humidity (>58.2%). Gold mine workers in the Mara region are increasingly susceptible to heat-related injuries and illness given the ambient temperature in this region can be greater than 30°C WBGT for extended periods.

Globally there are few available data on the contribution of occupational factors on the global burden of diseases [9], this situation has received little attention in Tanzania as well [10]. This study therefore aimed to create empirical evidence on whether hot environmental conditions contribute to heat-related illness so as to suggest effective strategies to enhance workers’ safety and health.

## Materials and Methods

### Study Population and Settings

A cross-sectional study was conducted at open cut and underground gold mines in the Mara region on May 2017. Miners with an employment record of more than one year and who had once requested treatment from the mine’s medical center for symptoms of heat-related illness were considered for inclusion while those who had shortly returned from leave and were not acclimatized were not included. Multistage sampling was used to select study subjects. At the first stage, workers were grouped into clusters of those who performed a specific task within similar thermal environmental exposure to form the Similar Exposure Group (SEG). Then two SEGs from open cut (quality controllers and offsiders) and two SEGs from the underground mine (jumbo offsiders and charge up crew) were selected to make four SEG groups. From the four selected SEGs, the Occupational Exposure Sampling Strategy Manual given by NIOSH was used to get the study subjects. A total of 60 mine workers were involved in the study. Eighteen jumbo offsiders and 19 service crew from underground the gold mine and 14 offsiders and nine quality controllers from the open cut gold mine were the selected workers from the four SEGs.

Work clothes were similar in all SEGs, the standard cotton long-sleeved shirt and pants. The shirt were 100% cotton with perforated reflective tape hoop pattern around the body, and had upper and lower back, underarm and front cooling vent. The shirts were yellow/navy and orange/navy in colors, representing workers from the main operator and contractors respectively. The navy color pants were also 100% cotton with a perforated reflective tape hoop pattern around the legs. Other work equipments such as gumboots, hard hats, hand gloves and safety glasses were similar for all workers.

### Study Instruments and Variable Definition

A preliminary evaluation of the work sections at gold mines was conducted during a walk-through survey. Information regarding heat stress control measures was collected using a developed checklist. An interviewer-administered questionnaire was used to obtain information from study subjects on the factors that influence heat stress. A closed-ended structured questionnaire was used as tool during data collection. We adapted the instrument from the Hunt et al., 2011 interviewer-administered questionnaire used in one of the gold mines in Australia. This was due to the similarities in approach and research topic.

The questionnaire comprised four sections. Section one addressed demographic information, including age, gender, height, weight and type of job/task performed. Section two focused on work environment and hydration practices, including frequency and volume of fluids consumption during work and the type of fluid consumption during break. In section three, study subjects were asked to indicate if they had experienced any symptoms of heat illness in the past 12 months (this included muscle cramp, fainting, headache, nausea, vomiting, weakness, fatigue, dizziness, clammy/moist skin, irritability, hot and dry skin, high body temperature, confusion, irrational behavior, low coordination, loss of consciousness and convulsions/seizures). These are the most common symptoms experienced during heat exhaustion and heat stroke. This list of symptoms was also in agreement with the conditions as outlined by the scientific and occupational hygiene communities, including the Australian Institute of Occupational Hygienist Heat Stress Standard and review articles on environmental thermal stress and industry. Other information on this section included the frequency of symptom occurrence as well as the work shift. Section four focused on information regarding medical conditions and prescribed medications.

Heat illnesses were classified as minor and moderate heat illness, based on the self-reported number of symptoms by the gold miner. Table 1 shows the symptoms of heat illness reported by miners. **Minor heat illness:** Experiencing more than once either less than four out eight heat exhaustion symptoms and/or less than three out of seven heat stroke symptoms. **Moderate heat illness:** Experiencing more than once either four out of eight heat exhaustion symptoms and/or three out of seven heat stroke symptoms. The ethical clearance was obtained from the Ethical Committee of the Muhimbili University of Health and Allied Sciences. Respondents were informed on the aim of the study for purposes of understanding and providing voluntary consent to participate in the study. Throughout the course of the study, confidentiality of the respondents was maintained.

**Table 1:** Symptoms of heat illness.

Symptoms of Heat Illness	Heat Illness

Painful spasm of muscle in the arms, legs or abdomen	Heat Cramps
Headache, nausea, vomiting, weakness and fatigue	Heat Exhaustion
Dizziness or light-headedness, Moist and pale skin	
Rapid heart rate and breathing, irritability	
High body temperature, hot and dry skin,	Heat Stroke
Confusion or disorientation, loss of consciousness	
Seizures, irrational behavior	

### Data Collection

Three physiological study parameters, which include core body temperature, pulse rate and blood pressure, were measured in each SEG. Data collection involved two measurements for each parameter, before work and eight hours after commencing work.

A digital clinical thermometer was used to measure core body temperature. The thermometer was placed under the tongue for two minutes before recording the temperature measurements. Each study subject had his/her own digital clinical thermometer.

The miners’ pulse rate was measured before the start of work and eight hours after the work shift. The automatic blood pressure monitor model SLD 3-107 of Suresign was used to measure both blood pressure (systolic and diastolic) and pulse rate simultaneously.

The waterless wet-bulb sensor and relative humidity sensor QUESTemp°46 was used to both measure and automatic calculate the dry-bulb, wet-bulb, globe, WBGT indoors, WBGT outdoors, relative humidity and heat index.

### Statistical Analysis

Categorical data were summarized as counts and percentages while continuous data were presented as means and standard deviation. The independent sample *t*-test and One Way ANOVA was used to determine the difference in mean environmental measures between surfaces and underground mines. Bivariate analysis and simple linear regression (Pearson co-relation coefficient [r] and coefficient of determination [r^2^]) were used to analyze the association between environmental measures and physiological measures. SPSS 20 (IBM) was used for all analysis.

## Results

### Descriptive Results

Demographic and anthropogenic characteristics of study participants are indicated in Tables 2 and 3. The majority of the study subjects were aged between 20–39 years (93.4%), most of them being male (91.7%) working as main operators (71.7%) and contractors (28.3%). Approximately 42% of them were normal weight and more than half of the miners were overweight: age (p-value = 0.512), height (p-value = 0.459), body mass (p-value = 0.058). BMI (p-value = 0.526) did not differ between gold miners working in open cut and underground.

**Table 2:** Demographic and anthropogenic characteristics of study participants.

Variables	Frequency	Percent (%)

**Gender**		

Male	55	91.7
Female	5	8.3
**Age Group (Years)**		

20–29	31	51.7
30–39	25	41.7
40–50	4	6.7
**BMI Group (kg/m^2^)**		

Underweight < 18.5	1	1.7
Normal 18.5–24.9	25	41.7
Overweight 25–29.5	31	51.7
Obesity > 30	3	5.0
**Employment Status**		

Main Operator	43	71.7
Contractor	17	28.3

**Table 3:** Demographic and anthropogenic characteristics of study participants working in underground and open cut.

Variables	Underground (n = 37)	Open Cut (n = 23)	*P*-Value

Age (years)	30.11 ± 6.41	30.52 ± 5.19	0.512
Height (m)	171.41 ± 8.51	173.83 ± 5.81	0.459
Body Mass (kg)	74.2 ± 6.7	76.1 ± 8.3	0.058
BMI (kg/m^2^)	25.4 ± 2.8	25.1 ± 2.2	0.526

### Proportion of Heat Illness Symptoms among Gold Mine Workers

Workers at open cut and underground goldmines reported at least one symptom of heat illness. Overall, high body temperature was the most frequently reported heat illness symptom (95%), followed by hot and dry skin (90%)as indicated in Table 4.

**Table 4:** Reported heat illness symptoms among open cut and underground miners.

Heat illness symptoms	Open cut (n = 23)	Underground (n = 37)	Chi-Square	*p*-value	Total (n = 60)

Muscle cramp	7 (30.4)	15 (40.5)	0.624	0.430	22 (36.7)
Headache	15 (65.2)	25 (65.6)	0.035	0.851	40 (66.7)
Nausea	2 (8.7)	0	–	–	2 (3.3)
Vomiting	2 (8.7)	0	–	–	2 (3.3)
Weakness	13 (56.7)	22 (59.5)	0.50	0.822	35 (58.3)
Fatigue	15 (65.2)	24 (64.9)	0.001	0.978	39 (65.0)
Dizziness	2 (8.7)	2 (5.4)	–	–	4 (6.7)
Moist skin	19 (82.6)	34 (91.9)	1.186	0.276	53 (88.3)
Irritability	7 (30.4)	13 (35.1)	0.141	0.707	20 (33.3)
Hot and dry skin	21 (91.3)	33 (89.2)	0.071	0.791	54 (90.0)
High body temperature	21 (91.3)	36 (97.3)	1.072	0.300	57 (95.0)
Confusion	6 (26.1)	13 (35.1)	1.212	0.271	19 (31.7)
Irrational behavior	8 (38.4)	7 (18.9)	1.904	0.168	15 (25.0)
Low coordination	1 (4.3)	1 (2.7)	–	–	2 (3.3)
Loss of consciousness	1 (4.3)	1 (2.7)	–	–	2 (3.3)

### Relationship between Heat Illness and Study Variables

Table 5 shows that working in underground and open cut (x^2^ = 0.588, *p* = 0.443), being jumbo offsiders, service crew or quality controllers (x^2^ = 0.055, *p* = 0.973), and employed under a main operator or contractor (x^2^ = 0.3.311, *p* = 0.069) had no effect on the proportion of the miners with moderate heat illness.

**Table 5:** Heat illness by potential study variables.

Study Variables	Heat Illness		Chi-Square	*p*-value

	Minor Heat Illness	Moderate Heat Illness		

**Mining site**				

Underground	8 (21.6)	29 (78.4)	0.588	0.443
Open cut	7 (30.4)	16 (69.6)		
**Job category**				

Jumbo offsiders	8 (25.0)	24 (75.0)	0.055	0.973
Charge up/service crew	5 (26.3)	14 (73.7)		
Quality controller	2 (22.2)	7 (77.8)		
**Employee’s status**				

Operators’ Employee	8 (18.6)	35 (81.4)	3.311	0.069
Contractors’ Employee	7 (41.2)	10 (58.8)		

### Association between Wet-Bulb Globe Temperature Index and Physiological Measures

From the independent sample *t*-test analysis (Table 6), the average wet-bulb globe temperature in open cut was higher (28.92 ± 1.87°C) compared to underground (27.09 ± 1.51°C). Similarly, average dry-bulb temperature in the open cut (31.9 ± 2.1°C) was higher than in the underground (30.1 ± 1.4°C). The difference in temperature values from both mine sites were statistically significant (p < 0.05).

**Table 6:** WBGT index and physiological measures by mining sites.

Parameters (n = 60)	Underground	Open cut	*p*- value	

	(n = 37)	(n = 23)		

Average wet-bulb globe temperature (°C)	27.09 ± 1.51	28.92 ± 1.87	0.000	*
Average dry-bulb temperature (°C)	30.1 ± 1.4	31.9 ± 2.1	0.000	*
Average relative humidity (%)	69.1 ± 7.8	40.72 ± 9	0.000	*
Average air velocity (m/s)	0.75 ± 0.25	1.74 ± 0.39	0.000	*
Core body temperature before (°C)	35.9 ± 1.7	36.5 ± 0.5	0.151	
Core body temperature after (°C)	37.3 ± 0.5	38.4 ± 0.5	0.000	*
Rise in core body temperature (°C)	1.1 ± 0.6	1.9 ± 0.8	0.000	*
Pulse rate before (beat/minute)	70.9 ± 14.6	81.35 ± 12.4	0.007	*
Pulse rate after (beat/minute)	94.7 ± 19.5	109.65 ± 15.9	0.003	*
Rise in pulse rate (beat/minute)	23.2 ± 15.3	28.30 ± 17.0	0.241	
Systolic blood pressure before (mmHg)	128.4 ± 11.7	129.3 ± 11.5	0.773	
Systolic blood pressure after (mmHg)	144.2 ± 13.8	149 ± 12.9	0.195	
Rise in systolic blood pressure (mmHg)	15.8 ± 8.4	19.7 ± 6	0.066	

*Significant at *p* < 0.05, Data summarizes mean ± SD.

The mean rise in core body temperature for workers in open cut was 1.9 ± 0.8°C while in underground was 1.1 ± 0.6°C. Mean rise in pulse rate for workers in open cut was 28.30 ± 17.0 beats/minute, while in underground was 23.2 ± 15.3 beats/minute. The independent sample *t*-test conducted showed that, for systolic blood pressure, the mean rise for open cut mine and underground workers were 19.7 ± 6mmHg 15.8 ± 8.4 mmHg respectively. The recorded changes of measured parameters in open cut were higher as compared to underground; however, the differences were significant only for the mean rise in core body temperature (*p* < 0.05). The air movement in the open cut mine was high as compared to the underground mine.

The results from one-way ANOVA (Table 7) indicated that the rise in core body temperature varied between the groups, with quality controller miners experiencing high rises in core body temperature (mean 1.81 ± 0.7°C). The difference on rise in core body temperature between job categories was found to be statistically significant (p < 0.05). The rise in pulse rate among jumbo offsiders was higher compared to the other job categories; however, the observed difference was not significant (p > 0.05). The variation in systolic blood pressure before work across the job category was statistically significant with quality controller miners having higher values (138.3 ± 7.9 mmHg).

**Table 7:** Physiological measures by job category.

Factors	Jumbo offsiders/offsiders (n = 32)	Charge up/service crew (n = 19)	Quality controller (n = 9)	F	*p*-value	

Core body temperature before (°C)	36.25 ± 0.5	35.8 ± 2.3	36.68 ± 0.69	1.283	0.285	
Core body temperature after (°C)	37.8 ± 0.7	37.34 ± 0.6	38.5 ± 0.5	9.510	0.000	*
Rise in core body temperature (°C)	1.63 ± 0.8	1.01 ± 0.5	1.81 ± 0.7	5.284	0.008	*
Pulse rate before (beats/min)	75.84 ± 14.4	66.78 ± 10.5	89 ± 11.7	9.119	0.000	*
Pulse rate after (beats/min)	104.18 ± 17.3	89.94 ± 20.1	109.5 ± 17.6	4.884	0.011	*
Rise in pulse rate (beats/min)	27.71 ± 14.6	23.15 ± 19.2	20.55 ± 13.6	0.920	0.404	
Systolic blood pressure before (mmHg)	127.34 ± 10.2	126.57 ± 13.3	138.3 ± 7.9	4.004	0.024	*
Systolic blood pressure after (mmHg)	143.4 ± 10.64	144.57 ± 16.04	158.88 ± 11.4	5.435	0.007	*
Rise in systolic blood pressure (mmHg)	16.06 ± 6.3	18 ± 9.9	20.55 ± 7.1	1.271	0.288	

**Figure 1:**
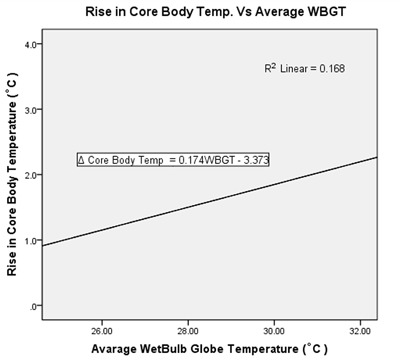
Correlation between WBGT and rise in core body temperature.

The relationship between wet-bulb globe temperature and average dry-bulb temperature with miners’ physiological parameters is indicated in Table 8. Bivariate analysis revealed the existence of positive correlation between them except for rise in pulse rate. The correlation between WBGT and rise in core body temperature was found to be significant with r = 0.410 and *p* = 0.001. Figure 1 shows a plot from linear regression analysis, which suggests that average WBGT predicts the rise in core body temperature by 16.8% (r^2^ = 0.168), and for a unit change of 1°C of average WBGT, then we predict a corresponding average change of 0.174°C of core body temperature.

**Table 8:** Association between environmental factors and physiological change.

Environmental factors	Rise in core body temperature	Rise in pulse rate	Rise in systolic blood pressure

Average dry-bulb temp	0.503* (*p* = 0.00)	0.006 (*p* = 0.962)	0.096 (*p* = 0.466)
Average WBGT	0.410* (*p* = 0.001)	–0.051 (*p* = 0.701)	0.033 (*p* = 0.801)

**Figure 2:**
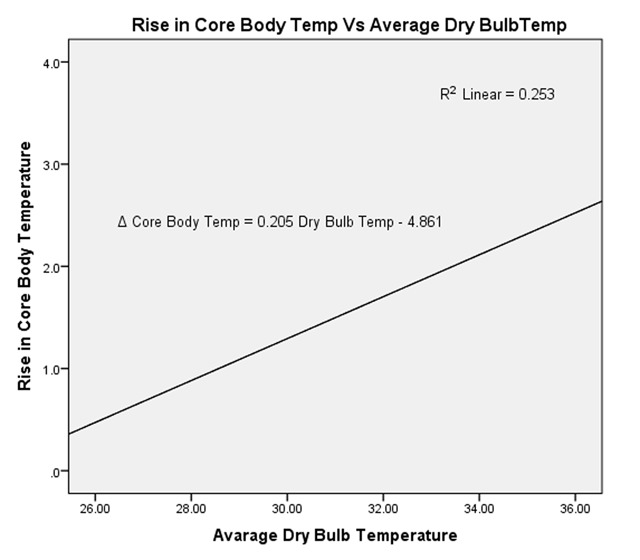
Correlation between dry-bulb temperature and rise in core body temperature.

A positive correlation (r) between dry-bulb temperature and rise in core body temperature, pulse rate and systolic blood pressure were r = 0.503, 0.006 and 0.096 respectively. The rise in core body temperature had a fair positive, statistically significant correlation with dry-bulb temperature (r = 0.503, *p* = 0.000). The coefficient of determination (r^2^ = 0.253) from Figure 2 indicates that the average dry-bulb temperature predicts the rise in core body temperature by 25.3%. For a unit change of 1°C of average dry-bulb temperature, then we predict a corresponding average change of 0.205°C of core body temperature.

### Hydration Practices and Characteristics

A majority, 41 (68.3%), had a free accessibility to safe and clean water (bottled water), whereas 19 (31.7%) could not get such access and had to either buy or drink the tap water at the workplace. Most of the workers (41.7%) do not drink water before commencing work, and only 35% take less than 250 mls, while 8% consume 250–500 mls. During a daily shift, more than half (56.7%) consume 3,000 mls of water, while 40% take greater than 3,000 mls. Overall the higher proportion of heat illness was observed among participants who did not drink any water prior to beginning of work shift, followed by those who consumed less than 250 mls as indicated in Table 9.

**Table 9:** Hydration practices among mine workers and heat illness.

Hydration characteristics	Frequency (%)	Chi-square	*p*-value	

Accessibility to free drinking water				
Yes	41 (68.3)	8.067	0.005	*
No	19 (31.7)			
Fluid intake before commencing work				
None	25 (41.7)	36.33	0.000	*
<250 ml	21 (35.0)			
250–500 mls	8 (13.3)			
500–1,000 mls	5 (8.3)			
>1,000 mls	1 (1.7)			
Fluid consumption during shift				
1,500 mls	2 (3.3)	26.800	0.000	*
3,000 mls	34 (56.7)			
>3,000 mls	24 (40.0)			
**Fluid intake before commencing work**	**Minor Heat illness**	**Moderate heat illness**	**Total**	

None	7 (28.0)	18 (72.0)	25 (41.7)	
<250 ml	6 (28.6)	15 (71.4)	21 (35.0)	
250–500 mls	0	8 (1000	8 (13)	
500–1,000 mls	2 (40)	3 (60)	5 (8.3)	
>1000mls	0	1 (1000)	1 (1.7)	

## Discussion

The findings of this study show that a majority of miners from both open cut and underground mines reported experiencing at least one symptom of heat illness. The prevalence of moderate heat illness was 78.4% for underground miners and 69.6% for open cut miners. This could be due to low air movement in underground mines, which was 0.75 ± 0.25 m/s compared to 1.74 ± 0.39 m/s in open cut.

Based on employer category, the proportion gold miners with moderate illness were 81.4% and 58.8% for those employed under operators and contractors respectively. This observation differs from the results obtained by Donoghue in 2004, who reported that miners under contractors had a higher proportion of heat illness compared to operators [11]. The difference could partially be because of gold miners’ need of money. Despite being provided with adequate and safe bottled water, workers under operators were observed to vend some of the bottled water to the nearby shops. This could increase their risk for dehydration and heat illness.

High body temperature is seen to be the most prevalent (98%) heat illness symptom, followed by hot and dry skin (90%). This finding is in agreement with the study done by Hunt et al., 2013, in Australian mining, where they reported high body temperature and hot and dry skin being among the most prevalent heat illness symptoms among miners [12].

The increase in average dry-bulb temperature and average wet-bulb globe temperature on the surface lead to the corresponding increase in the core body temperature of workers (r = 0.503: p < 0.01 and r = 0.410: p < 0.01 respectively). This result reveals the existence of an exposure-response relationship between the surface temperature and change in core body temperature. Similar observation was reported by Donoghue and Bates while determining the risk of heat exhaustion in relation to surface temperature at the underground mine [13]. Our results contrasted with the study done by Kalkowsky to determine the physiological strain of miners at hot working places in German mines, where he reported that heart rate and rectal temperature did not increase with climatic load [14]. The reasons for this dissimilarity could be due to the work-rest regime set by the administration within the two mine workplaces. In German mines, when climatic stress increases, workers are allowed to reduce their work load or take a work break and reduce their energy expenditure (self-pacing) to keep their sensation of strain at an appropriate level. The walk-through survey conducted during this study showed that the management did not schedule the short break for self-pacing during the working shift; open cut miners break once for lunch and it is during this time blasting was done, so they had at least a break of over one hour within the shift. Similarly, the underground miners break once for lunch but for a shorter time than the open cut miners.

Results from this study indicate that the overall averaged wet-bulb temperature in both open cut and underground gold mines was 27.7 ± 1.8°C. However, when the temperature was measured separately, data indicate the value of 27.09 ± 1.51°C for underground and 28.92 ± 1.87°C for open cut mine, respectively. The value of WBGT on underground operation were within the American Conference of Governmental Industrial Hygienists threshold limit value (ACGIH TLV) of 28°C for moderate work. For the open cut operation the averaged WBGT was above the ACGIH TLV, this could likely explain the observation of heat illness between the groups, where the proportions of miners with minor heat illness in underground and open cut were 21.6% and 30.4% respectively.

Moreover, the rise in core body for underground miners was 1.1 ± 0.6°C, while for open cut miners it was 1.9 ± 0.8°C, and the observed difference was statistically significant (p < 0.05). The present findings seem to be consistent with other studies [15,16,17], who reported the significance difference in WBGT index calculated for groups. Another important point to be noted is that, 78.3% of the open cut miners had the core body temperature above 38.0°C during their working shift. The core body temperature of 38.0°C is the ISO 7933 threshold for safety for acclimatized personnel. The finding is similar to the study by Hunt [18], where the core body temperature of more than 50% of the surface mine workers exceeded 38.0°C. Again, the existence of an exposure-response relationship between the surface temperature and change in core body temperature for open cut miners could likely be explained the Pearson correlation coefficient, which was fairly positive (r = 0.410).

The findings from this study indicated that 41.7% of workers do not drink water before commencing work while 35% take less than 250 mls. During the work shift, more than half of the workers consumed about 300 mls of water. This shows that majority of workers start the shift with low water consumption. Our finding is similar to the work of other researchers [18,19] who reported that many of the surface mine workers were dehydrated on commencing work and tended to remained so for the duration of the shift. Moreover, further results from this study indicated that the high proportion of heat illness was observed among miners who reported not consuming water prior to commencing work.

The study design being cross-sectional in nature does not give the causal effect analysis; thus we recommend a follow-up study on the association between hydration status of miners and accessibility to adequate safe water at all times. Further results in our study showed that 31.7% of workers did not have access to adequate and safe water; most of them were under a contractor employment scheme. They had access to treated tap water, but most perceived this to be unfit for consumption.

Several studies have indicated that the perceived taste of water influences hydration behavior. A study by Carter [20] on hydration knowledge, perception and behaviors, hydration status and needs reported that a majority of workers rated unfiltered water as tasting significantly worse than bottled water. To promote hydration behavior, the administration must be advised on providing water that is safe by regulatory standards and perceived as aesthetically fit by the consumers.

The significant difference in air circulation between the mining sites could be one of the causing factors of high prevalent moderate heat illness among workers in underground as compared to open cut. Ventilation in underground mines must be improved as the average air circulation was below 1.5 m/s.

The limitation of this study is that data collection was done only during one season of the year; the results can not reflect the annual ambient thermal condition at the mining site. Further investigation must be conducted throughout the year and with a larger group to improve the quality of the research, in particular regarding the possible association between heat stress symptoms and WBGT.

## Conclusions

The overall averaged wet-bulb globe temperature at the mining workplace was within the ACGIH TLV of 28°C; however, the mean core body temperature of open cut miners were above ISO 7933 threshold of 38°C for safety. Heat stress is a forgotten potential health problem in gold mines. This study indicates that high body temperature and hot and dry skin were among the most frequently reported heat illness symptoms. Moderate heat illnesses were also observed among underground and open cut miners. Despite the presence of hydration programs as an intervention to reduce development of heat-related illness, workers under contractors consumed less water prior to commencing work.

The results from this study recommend the following:

- Miners should be encouraged to report heat illness symptoms early and cease work when conditions worsen during a work shift.
- The OHS department should conduct periodic monitoring of the ambient thermal condition at the mining area and communicate the results to the workers and management.
- Miners should be informed on the detrimental health effects that may occur following hypohydration prior to the commencement of shift.
